# Prevalence, Antimicrobial Resistance, and Resistance Gene Profiles of Extended‐Spectrum β‐Lactamase‐Producing *Escherichia coli* and *Klebsiella pneumoniae* Isolated From Quails in Sylhet, Bangladesh

**DOI:** 10.1002/mbo3.70340

**Published:** 2026-06-16

**Authors:** Md. Al Mamun, Hemayet Hossain, Md. Shahidur Rahman Chowdhury, Shahida Begum Nargis, Munni Rani Das, Rakibul Hasan, Nasrin Akter Liza, Shuvojit Mitra, Md Bashir Uddin, Md. Mukter Hossain, Md. Mahfujur Rahman

**Affiliations:** ^1^ Department of Medicine Sylhet Agricultural University Sylhet Bangladesh; ^2^ Department of Veterinary Science and Animal Husbandry Teesta University Rangpur Bangladesh; ^3^ Department of Microbiology and Immunology Sylhet Agricultural University, Bangladesh Sylhet Bangladesh

**Keywords:** antimicrobial resistance, Bangladesh, *E. coli*, *Klebsiella pneumoniae*, multidrug resistance, quail

## Abstract

The rapid emergence of multidrug resistant Enterobacterales, especially extended‐spectrum β‐lactamase (ESBL)‐producing strains, poses a significant One Health challenge in low‐ and middle‐income countries, including Bangladesh. This study aimed to investigate the prevalence, antimicrobial resistance patterns, and resistant genes profile of *Escherichia coli* and *Klebsiella pneumoniae* isolated from quails in the Sylhet district of Bangladesh. A cross‐sectional study was conducted with 404 cloacal swabs were collected from quails across multiple retail locations. Isolates were identified using culture, biochemical tests, and PCR. Antimicrobial susceptibility was assessed by the Kirby–Bauer disk diffusion method against 12 antibiotics. Resistance and virulence genes, including *stx1*, *tetA*, *strA*, *AAC(3)‐iv*, *sul1*, and *β‐*lactamase genes, were detected using multiplex and monoplex PCR. Multiple antibiotic resistance index (MARI) and MDR status were determined. *E. coli* was detected in 63.37% (256/404) of samples, of which 53.91% were Shiga toxin‐producing (*stx1*). *K. pneumoniae* was confirmed in 11.14% (45) of isolates. Both organisms showed 100% resistance to ampicillin and amoxicillin–clavulanic acid. Nearly all isolates were multidrug resistant, with mean MARI values of 0.58 for *E. coli* and 0.48 for *K. pneumoniae*. High frequencies of resistance genes were observed, with strong phenotype–genotype alignment. In *K. pneumoniae*, the *bla*_TEM_ (65.96%), *Multicase*_MOX_ (44.68%) and *Multicase*_DHA_ (10.63%) genes predominated. Overall, the findings highlight quails in Bangladesh as an important and previously under recognized reservoir of multidrug resistant *E. coli* and *K. pneumoniae*, underscoring the urgent need for strengthened antimicrobial stewardship and One Health based surveillance strategies.

## Graphical abstract

This study investigated the molecular epidemiology and antimicrobial resistance of *Escherichia coli* and *Klebsiella pneumoniae* isolated from quails in Bangladesh. High prevalence of *E. coli*, including Shiga toxin‐producing strains, and widespread multidrug resistance were observed. Most isolates showed elevated MARI values and strong phenotype–genotype concordance, with frequent detection of resistance genes such as *tetA*, *strA*, *AAC(3)‐iv*, *blaTEM*, *MOX*, and *DHA*, highlighting significant One Health and food safety concerns.

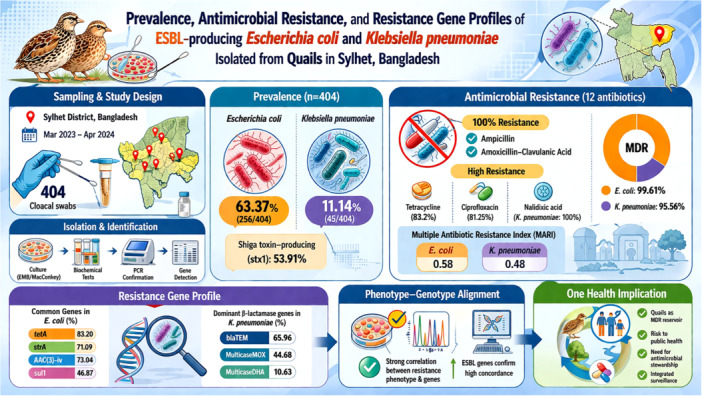

## 1 Introduction

The growing problem of antimicrobial resistance (AMR) is one of the biggest threats to modern medicine and the health of mankind, threatening the efficiency of medical treatments and adding to the number of infections being spread by diseases (Murray et al. 2022; Raquib et al. 2022). The World Health Organization emphasized a “One Health” approach, recognizing the close connection between human, animal, and environmental health in combating AMR (Woolhouse 2024). In Bangladesh, a rapidly growing poultry population, making a significant contribution to this country's economy, is being identified as a serious reservoir for the development of multidrug resistant (MDR) bacteria (Islam et al. 2023). The extensive use of antibiotics to promote growth and prevent disease in poultry drives bacterial resistance, enabling resistant bacteria to adapt and transfer from animals to humans through contaminated food (Neogi et al. 2020; Hossain et al. 2025). Among these, member of the Enterobacteriaceae family particularly *E. coli* and *K. pneumoniae* are the most prominent bacterial species contributing to human diseases (Hossain et al. 2026). *E. coli* is a commensal bacterium abundantly found in the intestines of warm‐blooded animals. However, it also includes a wide range of pathogenic variants that cause serious diseases in poultry as avian pathogenic *E. coli* (APEC) (Roy et al. 2025; Mandal et al. 2022), and in humans, such as urinary tract infections and septicemia (Mandal et al. 2021). *E. coli* is found abundantly within the poultry population of Bangladesh, reportedly above 60 percent, of which a significant proportion is MDR (Roy et al. 2025). Moreover, a virulent form, called Shiga‐toxin‐producing *E. coli*, or STEC, is a serious food‐borne pathogen (Rahman et al. 2024). STEC strains of this bacterium produce toxic proteins, namely Shiga‐toxins or *Stx1* and *Stx2*, that can cause colitis and the life‐threatening hemolytic‐uremic syndrome or HUS to human beings (Islam et al. 2008). The appearance of STEC among poultry and poultry products available for sale in Bangladesh reveals that direct zoonotic risks are being created through the handling of meats of these birds (Ali et al. 2025).

*K. pneumoniae*, is categorized under the ESKAPE group, identified frequently from chickens across Bangladesh (Khan et al. 2024). Although this bacterium is mainly associated with hospital infections, its increasing presence in reservoirs such as chickens is a serious concern due to its ability to acquire and spread resistance genes (Sultana et al. 2023). Both *E. coli* and *K. pneumoniae* have displayed a worrying trend of being able to produce Extended Spectrum Beta‐Lactamases (ESBL) (Hossain et al. 2026). These enzymes can degrade and render ineffective almost all beta‐lactam antibiotics, such as third‐generation cephalosporin antibiotics, that happen to be the last resort to combat infections from Gram‐negative bacteria effectively (Rahaman et al. 2025). Both *E. coli* and *K. pneumoniae*, found to be highly prevalent for producing ESBL, exposing the ineffectiveness predominantly of these most needed antimicrobial drugs, are being reported from Bangladesh (Amin et al. 2025; Zilon et al. 2026).

The *tetA* gene confers resistance to broad‐spectrum tetracycline antibiotics (Alam et al. 2023). *tetA* also encodes an efflux pump that codes for a protein responsible for expelling the antibiotics from a bacterial cell (Rafiq et al. 2024). *sul1* is often found within Class 1 integrons, which are mobile genetic elements that facilitate the co‐transfer of multiple resistance genes, thereby contributing significantly to MDR (Karim et al. 2023). *strA* typically encodes an aminoglycoside‐modifying enzyme that prevents the drug from binding to the bacterial ribosome, rendering it ineffective (Tanni et al. 2025). *aac‐3(iv)* also confers resistance to aminoglycosides, specifically through the production of an aminoglycoside acetyltransferase (Rafiq et al. 2022).

The high prevalence of multidrug resistance among the isolates of *E. coli* and *K. pneumoniae* in poultry of Bangladesh requires a better molecular understanding (Munim et al. 2024). However, unlike chickens and turkeys, numerous other birds that human beings consume have largely remained unexplored. Specifically, the rising trend of quail meat among bird meats has gradually become popular because of its lean nature, high immune system, and negligible fat content (Purohit et al. 2016). Currently, the Japanese quail is the most widely consumed quail for human food, found all across the world. Moreover, the fact that quail meat contains high protein, trace levels of harmful components, and high contents of minerals and vitamins makes them a ‘nutrient‐dense’ form of meat (López‐Pedrouso et al. 2019). But the microbiological safety of quail meat and its role as a potential vehicle for antimicrobial‐resistant bacteria are not as well‐documented. In addition, quails are sometimes reared closely together with other domestic animals and human populations, thus making them possible bridges for the transmission of ARGs between different species. However, the specific area of Sylhet City in Bangladesh was considered particularly relevant for this work because antibiotic use varied across regions, which could greatly affect the local resistome. Thus, the present study was conducted to determine the presence of *E. coli* and *K. pneumoniae* in quails in Sylhet City and to characterize their multidrug resistance phenotypes and key resistance genes.

## 2 Materials and Methods

### 2.1 Experimental Design and Sampling Approach

**Figure 1:**
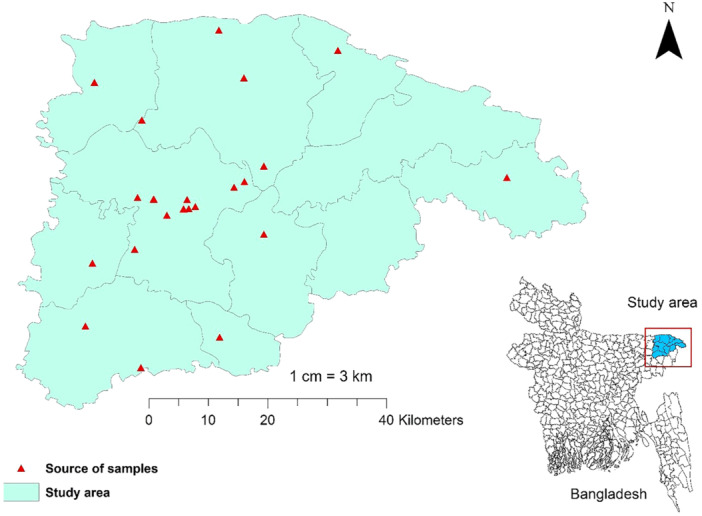
XY‐Coordination map showing the location of study area with specific location of sampling in Sylhet district of Bangladesh. The map was generated using ArcMap 10.8 software.

The cross‐sectional study was carried out in various places of Sylhet districts from which quail cloacal sample collection took place, including Tilagor point, Bondor Bazar, Shibganj point, South Surma Bazar, Horipur, Jaintapur, Pirer Bazar, Jokiganj Bazar, and some others. These areas lie in a geographic position between 24°36’ and 25°11’ Northern latitude and 91°38’ and 92°30’ Eastern longitude, respectively, as shown in Figure 1. Since there wasn't any study conducted in Sylhet districts, a sample size formula considering a prevalence of 50% prevalence, 95% confidence range, and accepted error percentage of 5% was used. Based on sample size determination, a minimum of 385 individual sample types would be needed. Finally, a total of 404 individual cloacal swabs were collected manually from the Quail. The target population needed to determine the prevalence was calculated from a standard formula (Rahman et al. 2024). Study samples were gathered using a random sampling technique between March 2023 and April 2024.

### 2.2 Specimen Collection and Microbiological Analysis

A total of 404 cloacal swab samples were collected from various retail establishments in the Sylhet district. Swabs were aseptically collected and stored in sterilized plastic bags along with Buffered Peptone Water (BPW; HiMedia Laboratories Pvt. Ltd., Mumbai, India). Samples were processed immediately after collection, with a delay of approximately 3–4 h (at 4°C) between collection and processing. A final concentration of 1:10 was followed in both swabs and BPW. The swabbed samples were incubated in this medium at 37°C for 24 ± 2 h. Enriched culture obtained from Buffered Peptone Water and an aliquot representing enriched culture in a loop were initially plated on EMB agar (HiMedia Laboratories Pvt. Ltd., Mumbai, India). All samples were incubated in this medium under aseptic conditions and in a way that samples were incubated in this medium at 37°C for a final duration of 24 ± 2 h (Basavaraju et al. 2022). The distinctive green metallic appearance was present in *E. coli*‐positive isolates in EMB agar. Following this process, EMB agar‐positive culture is incubated in pure culture in Nutrient Agar (NA) at 37°C for 24 ± 2 h. Biochemical tests were further conducted to ascertain confirmation (Smith 2003), including Gram staining, catalase, citrate, motility, indole, gas production, methyl red, urease, and triple sugar iron tests (Alizade et al. 2017).

MacConkey agar (HiMedia Laboratories Pvt. Ltd., Mumbai, India) was used to isolate and identify *Klebsiella* spp. For 24 to 48 h, the agar was incubated at 37°C (Elashkar et al. 2024). Confirmatory biochemical tests like gram staining, catalase test, citrate, motility, indole, gas production, methyl red, urease, and triple sugar iron test were employed (Sathyavathy and Madhusudhan 2021). Positive samples were prepared for PCR and genomic DNA extraction after these analyses.

### 2.3 Extraction of Genomic DNA

The conventional boiling technique was employed to isolate the genomic DNA from the *E. coli* and *Klebsiella pneumoniae* isolates as previously described by Aldous et al.(2005). The quality and quantity of purified DNA were analyzed by using the NanoDrop 2000c Spectrophotometer (Thermo Fisher Scientific Inc., USA). The monoplex PCR assay was developed for the detection of *alr, AAC(3)‐iv*, and *sul1* genes. Meanwhile, the multiplex PCR detection aimed to identify the *stx1, stx2, gyrA, rpoB, bla*_TEM_, *bla*_SHV_, *bla*_OXA_, *bla*_CTX‐M‐grp1_, *bla*_CTX‐M‐grp2_, *bla*_CTX‐M‐grp9_, *Multicase*_ACC_, *Multicase*_MOX_, *Multicase*_DHA_, *tetA*, and *strA* genes. Table 1 below indicates the sequences of the primers utilized.

**Table 1:** List of primers used in this study with annealing temperature.

Type of PCR	Primers (Gene)	Targeted Genes/Organism	Primer sequences	Amplicon size (bp)	Annealing Temp (°C)	Reference
UniPCR	*alr*	*Escherichia coli*	F‐ CTGGAAGAGGCTAGCCTGGACGAG	369	57	(Verbeke et al. 2014)
			R‐ AAAATCGCCACCGGTGGAGCGATC			
UniPCR	*Stx1*	*Shiga toxin producing E. coli*	F‐CGCTGAATGTCATTCGCTCTGC	302	55	(Memariani et al. 2015)
			R‐CGTGGTATAGCTACTGTCACC			
mPCR‐I	*gyr A*	*Klebsiella* spp.	F‐CGCGTACTATACGCCATGAACGTA	441	55	(Bobbadi et al. 2020; Chowdhury et al. (2025)
			R‐ACCGTTGATCACTTCGGTCAGG			
	*rpo B*	*K. pneumoniae*	F‐CAACGGTGTGGTTACTGACG	108	55	
			R‐TCTACGAAGTGGCCGTTTTC			
UniPCR	*peh X*	*K. oxytoca*	F‐GATACGGAGTATGCCTTTACGGTG	343	59	
			R‐TAGCCTTTATCAAGCGGATACTGG			
mPCR‐II	*bla*_TEM_	*TEM‐1 & 2*	F‐CATTTCCGTGTCGCCCTTATTC	800	62	(Dallenne et al. 2010)
			R‐CGTTCATCCATAGTTGCCTGAC			
	*bla*_SHV_	*SHV‐1*	F‐AGCCGCTTGAGCAAATTAAAC	713		
			R‐ATCCCGCAGATAAATCACCAC			
	*bla*_OXA_	*OXA‐1,4 & 30*	F‐GGCACCAGATTCAACTTTCAAG	564		
			R‐GACCCCAAGTTTCCTGTAAGTG			
	*bla*_CTX‐M1_	*CTX‐M‐1, CTX‐M‐3, & CTX‐M‐15*	F‐TTAGGAAATGTGCCGCTGTA	688		
			R‐CGATATCGTTGGTGGTACCAT			
	*bla*_CTX‐M2_	*CTX‐M‐2*	F‐CGTTAACGGCACGATGAC	404		
			R‐CGATATCGTTGGTGGTACCAT			
	*bla*_*CTX‐M9*_	*CTX‐M‐9 & CTX‐M‐14*	F‐TCAAGCCTGCCGATCTGGT	561		
			R‐TGATTCTCGCCGCTGAAG			
	*MultiCase*_ACC_	*ACC‐1 & ACC‐2*	F‐CACCTCCAGCGACTTGTTAC	346		
			R‐GTTAGCCAGCATCACGATCC			
	*MultiCase*_MOX_	*MOX‐1, MOX‐2, CMY‐1, CMY‐8 to CMY‐11 & CMY‐19*	F‐GCAACAACGACAATCCATCCT	895		
			R‐GGGATAGGCGTAACTCTCCCAA			
	*MultiCase*_*DHA*_	*DHA‐1 & DHA‐2*	F‐TGATGGCACAGCAGGATATTC	997		
			R‐GCTTTGACTCTTTCGGTATTCG			
mPCR‐III	*tet(A)*	Tetracycline	F‐GGCGGTCTTCTTCATCATGC	502	63	(Siddiky et al. 2021)
			R‐CGGCAGGCAGAGCAAGTAGA			
	*str(A)*	Streptomycin	F‐ATGGTGGACCCTAAAACTCT	893		
			R‐CGTCTAGGATCGAGACAAAG			
UniPCR	*AAC(3)‐iv*	Gentamicin	F‐AGTTGACCCAGGGCTGTCGC	333	58	(Heuer et al. 2002)
			R‐GTGTGCTGCTGGTCCACAGC			
UniPCR	*Sul1*	Sulfonamide	F‐CGGCGTGGGCTACCTGAACG	433	66	(Kozak, Boerlin, Janecko, Reid‐Smith, and Jardine, 2009)
			R‐GCCGATCGCGTGAAGTTCCG			

### 2.4 PCR‐Based Detection of Pathogens and Resistance Gene Profiling

Identification of *E. coli* was done by monoplex PCR to detect the *alr* gene, using extraction kits (Addbio Inc., Daejeon, South Korea). Resistance to gentamicin and sulfonamides was also done using monoplex PCR amplification of the *AAC(3)‐iv* and *sul1* genes, respectively. Moreover, multiplex PCR reactions were also carried out to amplify specific genes for the identification of the *Klebsiella* species (*gyrA*), *K. pneumoniae* (*rpoB*), Shiga toxin‐producing *E. coli* (*stx1 and stx2*), various ESBL genes, including *bla*_TEM_, *bla*_SHV_, *bla*_OXA_, *bla*_CTX‐M‐grp1_, *bla*_CTX‐M‐grp2_, *bla*_CTX‐M‐grp9_, *Multicase*_ACC_, *Multicase*_MOX_, *Multicase*_DHA_ and the *tetA* and *strA* genes for tetracycline and streptomycin resistance, respectively.

Reaction mixture and thermal cycling condition for molecular detection of different organisms were given in Table S1. The PCR products were confirmed using gel electrophoresis in a 1.8% or 1.5% low‐melting point agarose gel, and a 100 bp plus ladder was used to size the PCR products. Molecular‐grade nuclease‐free water was included as a negative control in each PCR assay to monitor contamination during amplification and gel electrophoresis. However, no positive control was included in the PCR assays.

### 2.5 Antimicrobial Susceptibility Testing (AST)

Kirby‐Bauer disk diffusion technique was employed for AST on Mueller‐Hinton agar (HiMedia Laboratories Pvt. Ltd., Mumbai, India) plates following the criteria set by the Clinical and Laboratory Standards Institute (CLSI) (CLSI 2020). Twelve antibiotics, classified into eight different antimicrobial class, included the following: penicillins (ampicillin 10 µg, amoxicillin‐clavulanic acid 20/10 µg), tetracyclines (tetracycline 30 µg), macrolides (azithromycin 15 µg), quinolones (ciprofloxacin 5 µg, nalidixic acid 30 µg), folate pathway inhibitors (trimethoprim‐sulfamethoxazole 1.25/23.75 µg), phenicol (chloramphenicol 30 µg), cephalosporins (ceftriaxone 30 µg), and aminoglycosides (gentamicin 10 µg, amikacin 30 µg, streptomycin 10 µg). The antibiotics were selected based on CLSI guidelines (CLSI 2020), previous studies (Islam et al. 2026; Chowdhury et al. 2025) and the field history of antibiotic use in Bangladesh. The bacterial suspension was obtained by choosing 3–5 well‐isolated colonies from an overnight culture, which was then suspended in normal saline to achieve the 0.5 McFarland turbidity standard (~ 1.5 × 10^8^ CFU/mL). The 0.5 McFarland turbidity standard was standardized according to the protocol used by (Hoque et al. 2023). The suspension was then equally distributed over the whole surface area of a Mueller‐Hinton agar (MHA) plate using a sterile cotton swab. After the surface had dried for a period of 3–5 min, the antibiotic discs were arranged with a minimum inter‐center distance of 24 mm. The plate was then incubated at a temperature of 37°C for a period of 16–18 h. The zone diameter was then measured using a ruler according to the CLSI breakpoints. Quality control for disk diffusion antimicrobial susceptibility testing was performed using *E. coli* ATCC 25922, and *K. pneumoniae* ATCC 13883 was included as a standard organism control strain, in accordance with CLSI guidelines. Each test was performed in triplets.

### 2.6 Double Disk Synergy Test (DDST)

Phenotypic detection of ESBL production was performed using the double disk synergy test. A bacterial suspension equivalent to 0.5 McFarland standard was inoculated onto MHA plates. An amoxicillin–clavulanic acid disk was placed at the center, and third‐generation cephalosporin disks (cefotaxime) were placed at a distance of 20–30 mm (center to center) from it. After incubation at 37°C for 18–24 h, enhancement of the inhibition zone of any cephalosporin disk toward the clavulanic acid disk (keyhole or synergy effect) was interpreted as positive for ESBL production.

### 2.7 Assessment of the Multiple Antibiotic Resistance (MAR) Index and MDR Distributions Among Bacterial Isolates

The MAR index was calculated using the formula below, which is described by Naser et al.(2024). MAR = (of antibiotics to which the isolate shows resistance)/(Total number of antibiotics tested). The MAR index is measured from 0 to 1. A lower MAR index indicates high susceptibility, and when closer to 1, the isolate is highly resistant. A high risk of bacterial contamination and resistance is established when the MAR is 0.20 and above. In addition, MDR is resistance to at least one agent in three antimicrobial classes or three antibiotic classes (Naser et al. 2024; Magiorakos et al. 2012).

### 2.8 Geospatial Mapping and Plotting

The shape file used to create the map with ArcMap 10.8 (ArcMap 10.8, Esri, USA) from www.diva-gis.org. Creating the dot map helps to effectively display the sample cluster of buffalo range in Sylhet, Bangladesh. A plot is created using GraphPad Prism 8.4. Geospatial mapping was used to visualize sampling distribution; however, no spatial clustering analysis of AMR patterns was performed.

### 2.9 Statistical Analysis

The data was analyzed using Excel spreadsheets in order to accurately organize the data. The chi‐square test was also done to test the association between the various explanatory variables. The confidence interval was also calculated using the binomial exact test, with a significance level set at *p* < 0.05. The SPSS software version 26 (SPSS Inc., Chicago, IL) was employed in the analysis.

### 2.10 Ethical Statement

The study was approved by the Institutional Ethics Committee of Sylhet Agricultural University, Sylhet‐3100, Bangladesh, under animal use protocol number #AUP2023022. All experimental procedures were conducted by trained professionals in strict compliance with the university's ethical guidelines and regulations. The welfare and well‐being of all animals involved in the study were prioritized and carefully maintained throughout the research.

## 3 Results

### 3.1 Prevalence of *E. Coli* and *K. pneumoniae*

Among 404 cloacal swab samples collected from quails, 256 isolates (63.36%) were confirmed as *E. coli*, while 94 isolates (23.27%) were identified as *Klebsiella* spp. PCR‐based confirmation revealed that 45 isolates (11.14%) belonged specifically to *K. pneumoniae* (Table 2). All molecularly confirmed isolates produced amplicons of expected sizes for *alr* (369 bp), *gyrA* (441 bp), and *rpoB* (108 bp), respectively (Figure S1).

**Table 2:** Prevalence of *E. coli* and *K. pneumoniae* isolated from fecal swab of quail in Sylhet district of Bangladesh.

Organisms	Positive isolates	Sample tested	% (95% CI)	Chi‐square value	*p*‐value
*E. coli*	256	404	63.37 (58.46–68.07)	302.57	< 0.001
*STEC*	138	256*	53.91 (47.59–60.13)		
*Klebsiella* spp.	94	404	23.27 (19.23–27.70)		
*K. pneumoniae*	45	404	11.14 (8.24–14.62)		

Abbreviations: CI, Confidence Interval; STEC, Shiga toxin producing *E. coli*.

* indicates frequency measures.

### 3.2 Detection of Shiga Toxin‐Producing *E. Coli*

Screening of all *E. coli* isolates for the *stx1* gene showed that 138 out of 256 isolates (53.91%; 95% CI: 47.59–60.13) were positive, confirming the presence of Shiga toxin‐producing *E. coli* (STEC) among quail samples.

### 3.3 Antimicrobial Susceptibility Phenotypes

**Figure 2:**
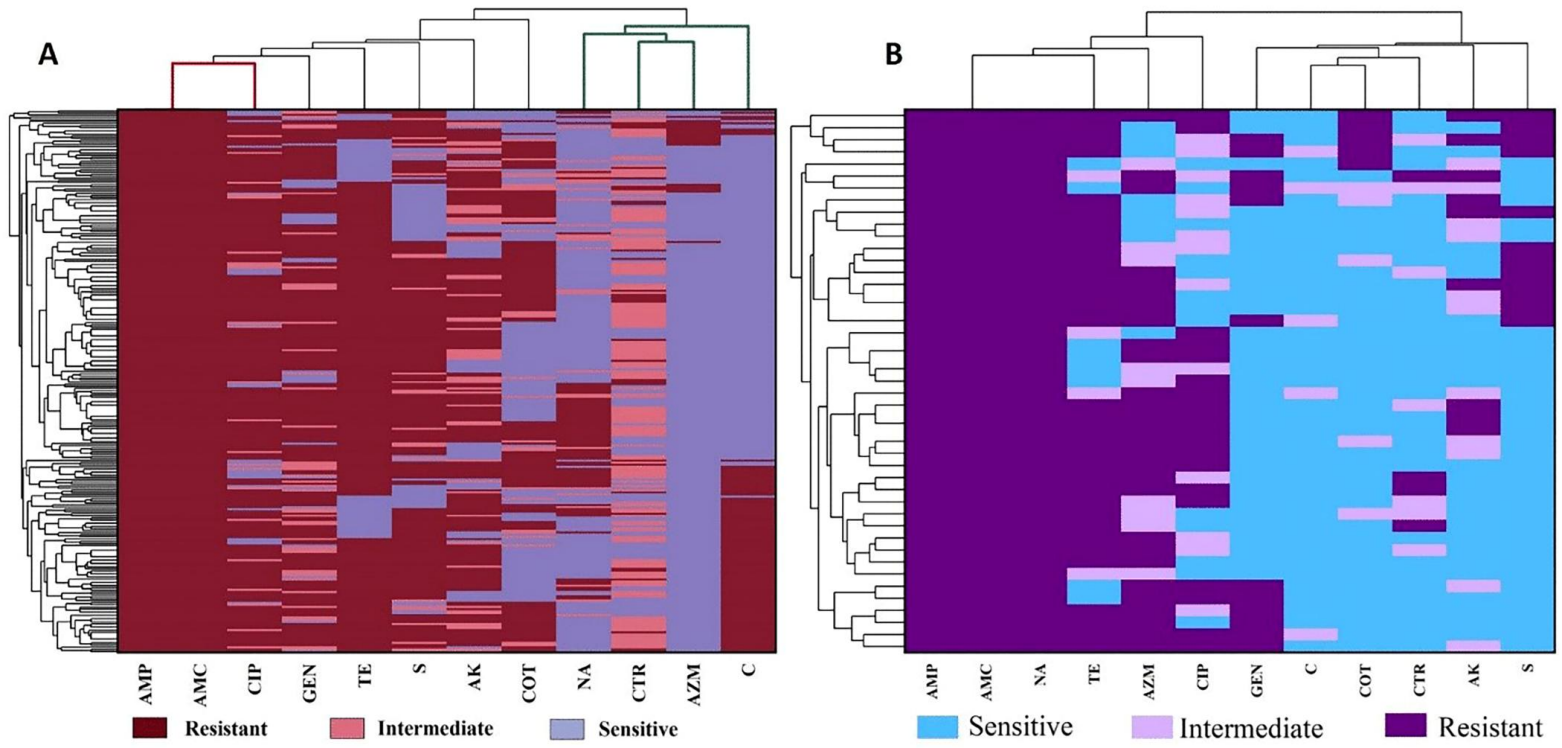
Heatmap with dendrogram showing the hierarchical cluster analysis among the selected antibiotics and also isolated samples. Hierarchical clustering showing similarity patterns in antimicrobial resistance profiles among isolates. Clustering reflects relationships between isolates and antibiotics based on resistance patterns. Figure (A) showing *E. coli* isolates and (B) showing *K. pneumoniae* isolates.

**Figure 3:**
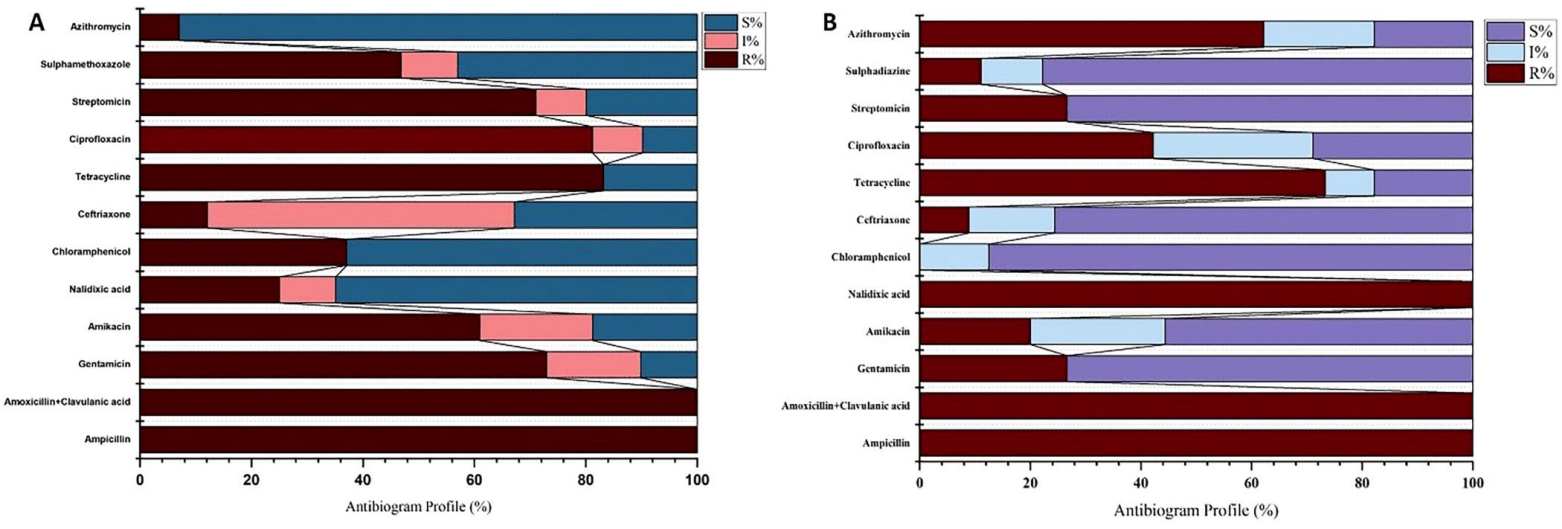
Antibiogram profile showing the percent of resistance, sensitive and intermediate among the isolates of *E. coli*. (A) and *K. pneumoniae*. (B) on quail in Sylhet region.

All *E. coli* and *K. pneumoniae* isolates exhibited resistance to multiple antimicrobial agents (Figures 2 and 3). Complete resistance (100%) to ampicillin and amoxicillin–clavulanic acid was observed in both organisms. High resistance among *E. coli* isolates were recorded for tetracycline (83.20%) and ciprofloxacin (81.25%), whereas *K. pneumoniae* showed complete resistance (100%) to nalidixic acid, ampicillin and amoxicillin–clavulanic acid. Conversely, higher susceptibility was observed for azithromycin (92.96%) in *E. coli* and chloramphenicol (87.50%) and sulphamethoxazole–trimethoprim (77.78%) in *K. pneumoniae*.

### 3.4 Detection of Antimicrobial Resistance Genes

**Figure 4:**
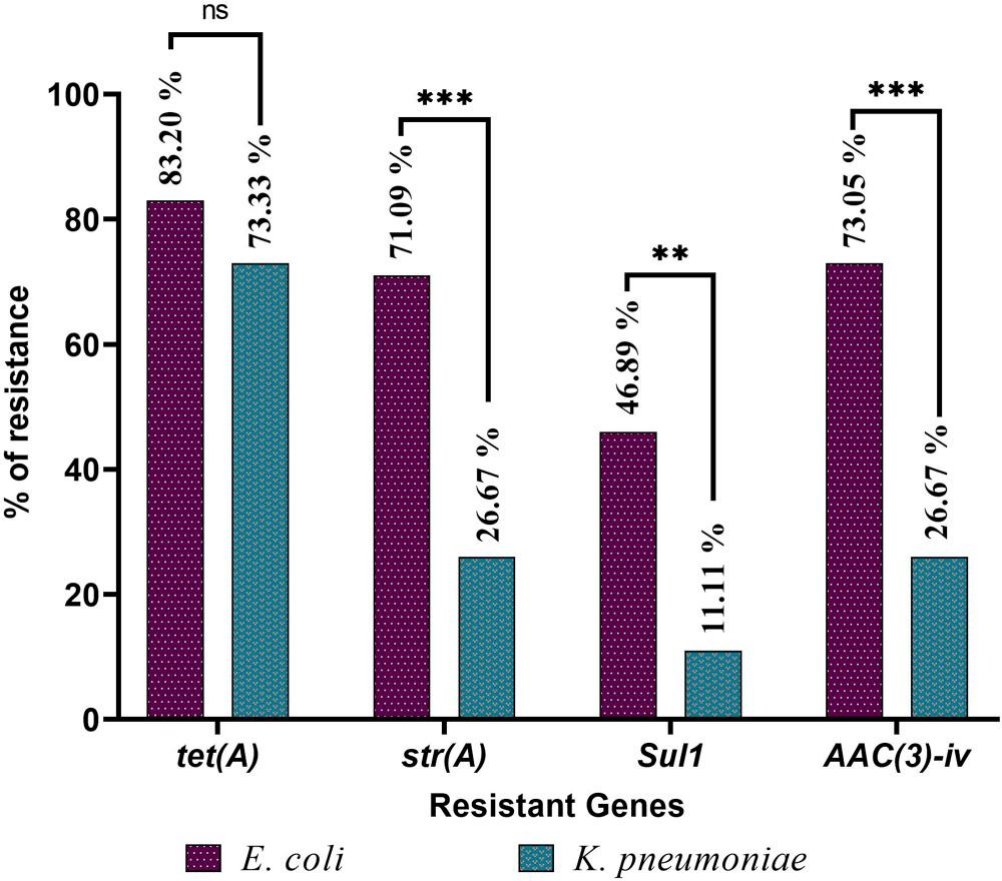
Genotypic frequency of antimicrobials resistant genes on *E. coli* and *K. pneumoniae* isolated from cloacal swab of quail in Sylhet. ns: non‐significant, *p < 0.05, **p < 0.01, ***p < 0.001.

PCR‐based screening of resistance genes revealed a high prevalence of genotypic resistance in both organisms. Among *E. coli* isolates, the *tetA* gene was detected in 83.20%, followed by *strA* (71.09%), *AAC(3)‐iv* (73.04%), and *sul1* (46.87%). In *K. pneumoniae*, resistance genes were detected at comparatively lower frequencies, with *AAC(3)‐iv* present in 73.33%, followed by *tetA* (26.66%), *strA* (26.66%), and *sul1* (11.11%) of isolates (Figure S2, Figure 4).

### 3.5 Phenotype‐Genotype Correlation

**Figure 5:**
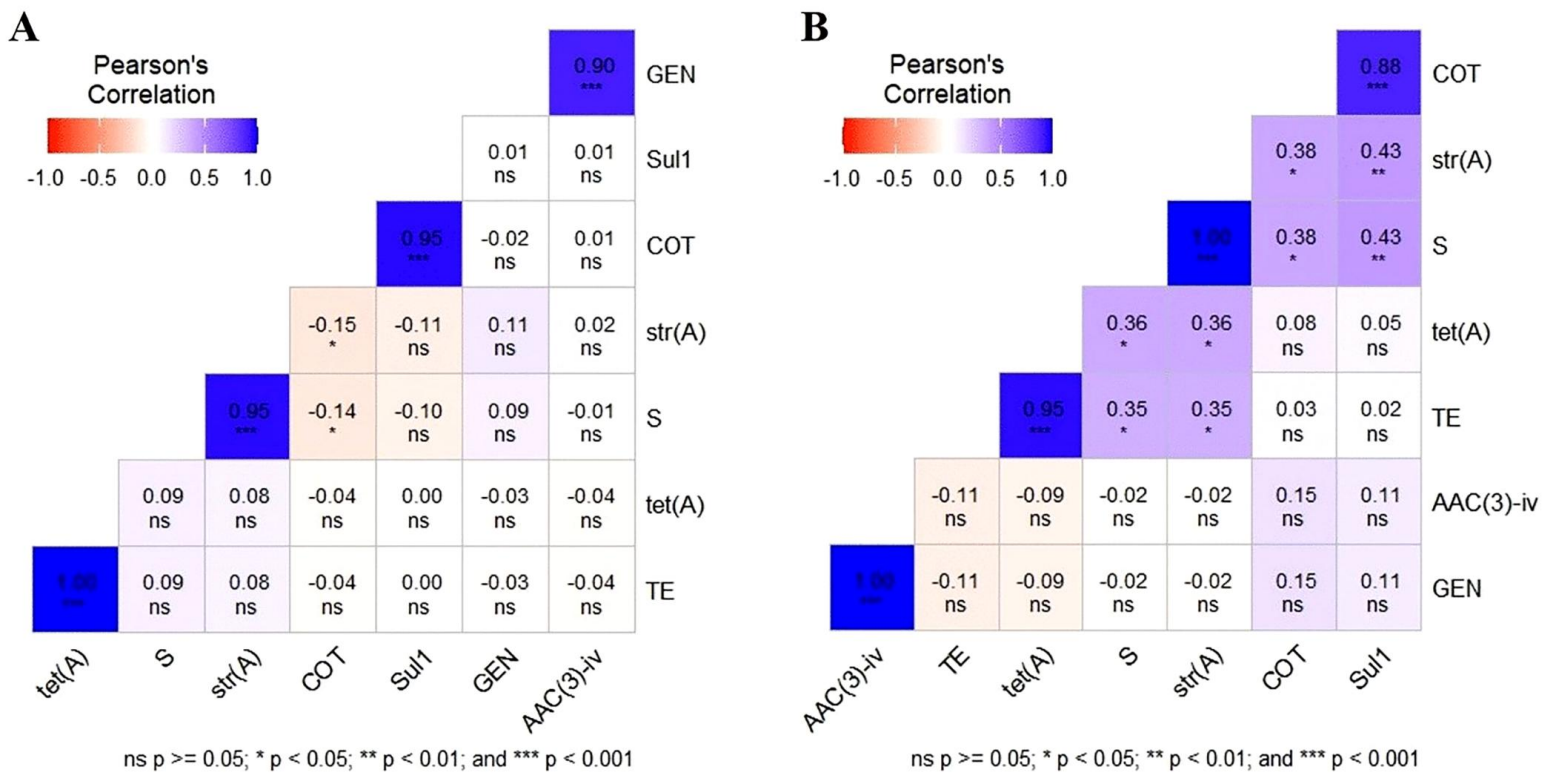
Genotype‐phenotype correlation among *E. coli* isolates. (A) and *K. pneumoniae* isolates. (B) of the mostly used antibiotics on quail in Sylhet region.

Pearson's correlation analysis demonstrated strong and significant genotype–phenotype concordance for antimicrobial resistance. In *E. coli*, tetracycline resistance showed a perfect correlation with *tet(A)* (*r* = 1.00, *p* < 0.001), while streptomycin resistance was strongly associated with *str(A)* (*r* = 0.95, *p* < 0.001); sulfonamide resistance also correlated with *sul1* (*r *= 0.90, *p* < 0.001). Similarly, in *K. pneumoniae*, tetracycline and streptomycin resistance were highly correlated with *tet(A)* (*r* = 1.00, *p* < 0.001) and *str(A)* (*r* = 0.95, *p* < 0.001), respectively. Sulfamethoxazole‐Trimethoprim resistance showed a moderate association with *sul1* (*r* = 0.68, *p* < 0.01), whereas *aac(3)‐IV* exhibited weak or non‐significant correlations (Figure 5).

### 3.6 Multidrug Resistance Index and MDR Status

The multiple antibiotic resistance index (MARI) analysis demonstrated a high antimicrobial exposure risk (Table 3). *E. coli* isolates exhibited a mean MARI value of 0.58 ± 0.10, while *K. pneumoniae* showed a mean MARI of 0.48 ± 0.11.

**Table 3:** Multiple antibiotic resistance index (MARI) and multidrug resistance profile of isolated *E. coli* and *K. pneumoniae* from cloacal swab of Quail.

Organisms	MARI (Mean ± SD)	Sig.	MDR (%)	95% CI	Sig.
*E. coli*	0.58 ± 0.10	ns	99.61 (255/256)	97.84‐99.99	ns
*K. pneumoniae*	0.48 ± 0.11		95.56 (43/45)	84.85‐99.46	

Abbreviations: CI, confidence interval; ns, non‐significant; SD, standard deviation; Sig., significance level.

Nearly all *E. coli* isolates (99.61%; 95% CI: 97.84–99.99) and 95.56% (95% CI: 84.85–99.46) of *K. pneumoniae* isolates were classified as multidrug‐resistant (Table 4). No statistically significant difference was observed between the organisms for MDR prevalence (*p* > 0.05).

**Table 4:** The frequency of *β*‐lactamase genes among the positive isolates of *K. pneumoniae* (*n* = 94) and *E. coli* (*n* = 256) from fecal swab of Quail in Sylhet District.

β‐lactamase genes	*K. pneumoniae*		*E. coli*	
	Frequency	% (95% CI)	Frequency	% (95% CI)
*bla*_TEM_	62	65.96 (55.46–75.42)	125	48.83 (42.55–55.13)
*bla*_SHV_	0	0	53	20.70 (15.91–26.19)
*bla*_OXA_	9	9.57 (4.47–17.40)	37	14.45 (10.39–19.37)
*bla*_CTX‐M‐group1_	0	0	0	0
*bla*_CTX‐M‐group2_	0	0	0	0
*bla*_CTX‐M‐group9_	0	0	0	0
*MultiCase*_ACC_	0	0	0	0
*MultiCase*_MOX_	42	44.68 (34.41–55.29)	0	0
*MultiCase*_DHA_	10	10.63 (5.22‐18.70)	0	0

Abbreviations: CI, confidence interval; N/A, Not applicable.

### 3.7 Detection of ESBL‐Encoding Genes

Multiplex PCR screening of β‐lactamase genes among *K. pneumoniae* isolates (*n* = 94) revealed a significant presence of *β*‐lactamase genes (*p* < 0.001). Phenotypically, a total of 53 (56.38%) isolates showed enhanced zone (key hole) indicated as ESBL producer. The *bla*_TEM_ gene was detected in 62 isolates (65.96%) followed by *MultiCase*_MOX_ in 42 isolates (44.68%) and *MultiCase*_DHA_ in 10 isolates (10.63%). The *bla*_OXA_ gene was detected in 9 isolates (9.57%), whereas *bla*_SHV_, *bla*_CTX‐M‐group1_, *bla*_CTX‐M‐group2_, *bla*_CTX‐M‐group9_, and *MultiCase*_ACC_ were not detected in any isolate (Table 4). Among the *E. coli* isolates (*n* = 256) recovered from quail fecal swabs, *bla*_TEM_ was the most prevalent β‐lactamase gene, detected in 125 isolates (48.83%, 95% CI: 42.55–55.13). This was followed by *bla*_SHV_, identified in 53 isolates (20.70%, 95% CI: 15.91–26.19), and *bla*_OXA_, detected in 37 isolates (14.45%, 95% CI: 10.39–19.37). In contrast, no *E. coli* isolates harbored *bla*_CTX‐M‐group1_, *bla*_CTX‐M‐group2_ or *bla*_CTX‐M‐group9_ genes, nor were AmpC β‐lactamase genes (*ACC, MOX, or DHA*) detected.

## 4 Discussion

The present study demonstrated a high prevalence of *E. coli* (63.37%) and *K. pneumoniae* (11.14%) among quail cloacal swabs in Sylhet, Bangladesh. Notably, 53.91% of *E. coli* isolates were identified as Shiga toxin–producing. Furthermore, multidrug resistance was observed in 99.61% of *E. coli* and 95.56% of *K. pneumoniae* isolates, with mean MARI values of 0.58 and 0.48, respectively. These findings indicate a substantial burden of antimicrobial‐resistant Enterobacterales in quails, highlighting their potential role as a zoonotic reservoir.

The prevalence of *E. coli* in cloacal swabs was relatively high, indicating substantial intestinal colonization. However, this value was lower than those reported in previous studies on quail fecal and cloacal samples (82.2% and 83%, respectively) (Al‐Amin et al. 2022; Wahid et al. 2019). These variations may be attributed to differences in sampling techniques, geographical location, farm management practices, and the health status of the birds. Additionally, although the current prevalence is lower than some reports from other avian populations and *E. coli* pathotypes, it remains higher than findings reported in certain poultry studies (Ali et al. 2025; Zilon et al. 2026).

Analysis of the Shiga toxin producing gene (*stx1*) found that 53.91% of the isolates were positive. This is significantly higher than the prevalence found amongst quails from other geographical parts of the world, which ranges from 5.7% to 20% (Dipineto et al. 2014; El–dayem et al. 2020). Though high rates of STEC have been found within other domesticated farm animals, such as cows and goats, in Bangladesh (Islam et al. 2008), the rate observed in quails in Sylhet has one of the highest rates to be found within the literature. The detection of the *stx1* gene is extremely concerning because the prokaryotic STEC is the direct cause of serious human infections such as hemorrhagic colitis, which further leads to the potentially deadly Hemolytic Uremic Syndrome if left untreated (Chiacchio et al. 2016).

In addition, the finding of *Klebsiella* spp. from the cloacal swabs of quails, with confirmation of *K. pneumoniae* of 11.14% from the isolates, also suggests the carrier status of the quails with potential Enterobacterales. This percentage almost agrees with the previous studies from the cloacal swabs of other bird species, such as 10% from the isolates of poultry (Putri et al. 2026) and 11.67% from the duck cloacal swabs (Thesia et al. 2025). However, the prevalence found in the current study seems lower when compared with that of broiler chicken intestinal swabs from Iraq (29.5%) (Musa 2025) and as well as swabs of chicken meat from Bangladesh (34.74%) (Tanni et al. 2025). The cloacal swabs would more accurately depict the intestinal prevalence of the bacteria. On the contrary, intestinal swabs as well as meat swabs might vary as a result of slaughtering practices. Additionally, host species differences, farm management practices, geographic factors, and methodological variations may further contribute to discrepancies in reported prevalence across studies.

A study from Turkey reported approximately 93% susceptibility of *E. coli* isolates from quail cloacal swabs to this antimicrobial, indicating substantial variation in β‐lactam resistance (Buyukunal et al. 2019). Similarly, tetracycline exhibited the highest resistance in the present study, which contrasts with findings from Egypt where *E. coli* showed high susceptibility to these antimicrobials (Etman et al. 2023). However, our results are consistent with reports from Ethiopia, where high resistance to ampicillin and tetracycline among poultry *E. coli* isolates has been documented, likely reflecting selective pressure from widespread antimicrobial use (Shecho et al. 2017). Ciprofloxacin showed a high resistance rate (81.25%), indicating reduced therapeutic effectiveness, whereas 100% susceptibility was reported in Ethiopian poultry isolates under similar conditions (Shecho et al. 2017).

Amoxicillin/Clavulanic acid showed high resistance, consistent with earlier studies conducted on duck cloacal swab samples in Indonesia and quail meat samples in Egypt (Younis et al. 2021), hence, this underlined the waning effectiveness of this antibiotic against *K. pneumoniae* in birds (Kahin et al. 2024).

Data on the frequency of ARGs *in E. coli* isolates in the current study shows similarity with previous reports in Bangladesh. It is essential to note that *tetA* has the highest frequency of the detected gene in quail‐associated *E. coli* isolates. The extensive and often unregulated use of tetracyclines in poultry production systems likely exerts strong selective pressure, facilitating the persistence of resistance genes such as *tetA*. In contrast, lower resistance rates reported in some studies may reflect stricter regulations on antibiotic use, improved biosecurity, and differences in veterinary oversight.

Its frequency is in close agreement with those detected in the marketing sample of the broiler meat (84%) and chicken eggs (82.22%) in Bangladesh (Alam et al. 2023; Sultana et al. 2023). However, it has the highest frequency compared with the previous reports detected in the cloacal swabs of the same species of bird, implying a relatively greater selection pressure in the quail‐associated microbial ecological niches (Sarker et al. 2019; Roberts 2005; Alam et al. 2023).

The geographic variation in antimicrobial use practices, differences in farm hygiene, and variations in bacterial population dynamics may further contribute to the observed differences.

Moreover, the frequency of ARGs detection is substantially higher compared with those detected in the blood, liver, and bone marrow of the commercial chickens (broilers, layers, and breeders), in which the detection rates were relatively lower (Bhattarai et al. 2024). In fact, this discrepancy can partly be explained by the increased frequency of HGT in the gastrointestinal tract, particularly in the cloacal environment under the continuous influence of antimicrobial selection pressures. On the contrary, *sul1* has the least frequency of detection among the detected antimicrobial resistance in *E. coli* isolates in the current study. Surprisingly, its frequency level is modestly higher compared with the study with similar characteristics, in which the gene *sul1* was found in only 20.1% of the isolates (Bhattarai et al. 2024). This indicates a gradual but notable expansion of sulfonamide resistance within quail‐associated *E. coli* populations.

A distinct susceptibility gene profile in *K. pneumoniae* revealed that *AAC‐3(iv)* gene is the dominant gene, with the highest level of resistance, occurring in this bacterial species in this research. On the other hand, *strA, tetA*, and *sul1* have lower frequencies of occurrence, which constitute the lowest prevalence level altogether. Contrary to the research findings presented in a study in Sylhet, Bangladesh, in a poultry meat source, the *tetA* and *sul1* gene prevalence is over 80%, while *AAC‐3(iv)* is present in a minimal level of 7.07% (Tanni et al. 2025; Garneau‐Tsodikova et al. 2015).

In *K. pneumoniae*, *AAC*(3)‐IV was detected in 73.33% of gentamicin‐resistant isolates, indicating shared aminoglycoside resistance mechanisms with *E. coli* in the avian gut environment. However, only 26.66% of tetracycline‐ and streptomycin‐resistant isolates harbored *tetA* and *strA*, respectively, implying a more complex resistance repertoire in *K. pneumoniae*, potentially involving alternative genes (e.g., *tetB, aadA*) or non‐enzymatic mechanisms. Overall, the > 70% phenotype–genotype agreement for aminoglycoside and tetracycline resistance in both organisms supports the use of targeted molecular assays for AMR surveillance (Messele et al. 2022).

The prevalence of MDR‐*E. coli* isolates in the present study was exceptionally high, with 99.61% of cloacal isolates from quail, representing one of the highest MDR burdens reported to date for quail‐associated *E. coli*. In contrast, the percentage of *E. coli* isolates that were MDR in quail cloacal swabs in Indonesia was significantly lower as it was 18% (Rahayu et al. 2025). Similarly, the percentage of MDR in quail cloacal swab samples in Egypt (54.67%) was found to be lower compared to the relatively high percentages of MDR discovered in the current study (Etman et al. 2023). On the other hand, the current study is in agreement with the study in Bangladesh, in which all *E. coli* isolates obtained from the cloacal swab samples of broilers were found to be MDR (Sarker et al. 2019). The similar percentage of MDR found in these studies reveals that the intestinal *E. coli* in intensive poultry production systems, irrespective of species, remain under intense antimicrobial selection pressure, leading to the development of MDR phenotypes.

A similar resistance pattern was also noted in *K. pneumoniae*. In a recently conducted study involving samples of broiler meats, a 100% rate of MDR was recorded in *K. pneumoniae* isolates, and MAR indices varied between 0.33 and 0.83 with a mean of 0.57 (Tanni et al. 2025). These findings closely mirror the high MDR prevalence and elevated MARI values recorded in the present study, reinforcing the widespread nature of multidrug resistance in *K. pneumoniae* across poultry‐associated matrices. However, the MDR burden and MARI values observed here were somewhat higher than those reported from broiler cloacal swabs and environmental samples in China (Li et al. 2022) as well as from duck cloacal swabs in Indonesia, where 76.19% of *K*. *pneumoniae* isolates were MDR (Thesia et al. 2025).

Furthermore, 45.45% and 68.18% of *E. coli* isolates from quail cloacal swabs were found to carry *bla*_*CTX‐M*_ and *bla*_*TEM*_ genes in a study in India, which is in agreement with the present study's findings (Sakthikarthikeyan et al. 2024). In another study in Bangladesh, from broiler cloacal swabs *bla*_*TEM*_ was found 75.67% which was also near to the findings of present study (Sarker et al. 2019). Besides, in case of *K*. *pneumoniae* isolates from quail cloacal swabs revealed a significant burden of β‐lactamase genes (*p* < 0.001), supporting the phenotypic resistance patterns observed. *bla*_*TEM*_ was the dominant determinant, detected in 65.96% of isolates, consistent with its widespread plasmid‐mediated dissemination in poultry‐associated *K. pneumoniae* and sustained selection by penicillin and early‐generation cephalosporins (Paterson and Bonomo 2005; Nordmann et al. 2011; Veloo et al. 2022). The predominance of *bla*_TEM_ suggests widespread plasmid‐mediated resistance, while AmpC genes (*MOX, DHA*) indicate additional β‐lactam resistance mechanisms beyond ESBL production.

AmpC‐type β‐lactamases were also prominent, with *MultiCase*_*MOX*_ (44.68%) and *MultiCase*_*DHA*_ (10.63%), indicating substantial non‐ESBL β‐lactam resistance capable of conferring reduced susceptibility to cephamycins and inhibitor‐protected β‐lactams (Jacoby 2009). Similarly, AmpC prevalence has been reported in poultry‐derived *K. pneumoniae* from Asia, suggesting an expanding role of AmpC enzymes in avian reservoirs (Roberts et al. 2024; Thesia et al. 2025). In contrast, *bla*_*OXA*_ was detected at a low frequency (9.57%), aligning with reports that OXA‐type enzymes are less common in poultry than in clinical settings (Smet et al. 2010) Notably, *bla*_*SHV*_, *bla*_*CTX‐M*_ groups 1, 2, and 9, and *MultiCase*_*ACC*_ were absent altogether, suggesting the absence of CTX‐M ESBL‐types among this group of quails. This contrasts with studies in broiler meat and environmental samples where CTX‐M enzymes predominate (Cardozo et al. 2021; Mahanti et al. 2018).

Variability in resistance patterns across studies may also arise from methodological differences, including sample types (cloacal swabs vs meat samples), study design, and laboratory protocols. Furthermore, environmental factors, such as local antimicrobial contamination and horizontal gene transfer within microbial communities, may influence the distribution of resistance genes across different regions.

Given the high prevalence of multidrug resistance observed, there is an urgent need to strengthen antimicrobial stewardship in the poultry sector of Bangladesh. Practical interventions should include the implementation of stricter regulations on antibiotic use, particularly limiting non‐therapeutic applications such as growth promotion and prophylaxis. Establishing routine AMR surveillance programs across different poultry production systems is essential to monitor emerging resistance trends. In addition, farmer education and training programs should be promoted to encourage minimal antibiotic use, improved farm hygiene, and biosecurity practices. The adoption of alternative strategies, such as probiotics, vaccination, and improved nutrition, may further reduce reliance on antibiotics and mitigate the spread of antimicrobial resistance. Collectively, these measures are critical to safeguarding both animal and public health within a One Health framework.

### 4.1 Strengths and Limitations

This study is among the first to molecularly characterize multidrug‐resistant *E. coli* and *K. pneumoniae* from quails in Bangladesh. A large sample size and multi‐site sampling enhance representativeness. Combined phenotypic and genotypic analyses strengthen resistance profiling and phenotype–genotype concordance, providing important One Health–relevant evidence. This study has several limitations. Only limited virulence genes were investigated, with detection restricted to *stx1*, while other important STEC‐associated genes such as *stx2* and *eae* were not included. Additionally, plasmid characterization and mobile genetic element analysis were not performed, which limits understanding of horizontal gene transfer dynamics. Sampling was limited to retail‐level quails in Sylhet and may not represent farm‐level variation. Information on farm type (commercial vs backyard) and antimicrobial usage was not available. Sampling from a single region may restrict generalizability. Lack of whole‐genome sequencing and antimicrobial usage data constrains understanding of transmission dynamics and resistance drivers.

## 5 Conclusion

This study demonstrates a high prevalence of multidrug‐resistant *E. coli* and *K. pneumoniae* in quails from the Sylhet district of Bangladesh, highlighting quails as an important and previously under recognized reservoir of antimicrobial‐resistant bacteria. The detection of a substantial proportion of Shiga toxin‐producing *E. coli*, together with widespread multidrug resistance and elevated MARI values, underscores a significant zoonotic and food safety risk. Strong concordance between phenotypic resistance and resistance genes, including the dominance of *tetA*, *strA*, *AAC(3)‐iv*, and β‐lactamase genes such as *bla*_TEM_, *bla*_MOX_, and *bla*_DHA_, indicates sustained antimicrobial selection pressure within quail production systems. These findings emphasize the need to extend antimicrobial resistance surveillance beyond conventional poultry species and incorporate quails into national monitoring frameworks. Implementation of prudent antimicrobial use policies, improved biosecurity, and routine molecular surveillance is essential to limit further dissemination of resistant pathogens. Adopting a One Health–based approach integrating animal, human, and environmental health sectors will be critical to mitigate the public health threat posed by antimicrobial resistance originating from alternative poultry sources.

## Author Contributions

**Md. Al Mamun:** data curation, investigation, formal analysis, methodology, software, writing – original draft; writing – review and editing. **Hemayet Hossain:** data curation, investigation, formal analysis, methodology, software, writing – original draft, writing – review & editing. **Md. Shahidur Rahman Chowdhury:** data curation, investigation, formal analysis, methodology, software, writing – original draft; writing – review and editing. **Shahida Begum Nargis:** investigation, methodology. **Munni Das:** investigation, methodology. **Rakibul Hasan:** investigation, methodology. **Nasrin Akter Liza:** investigation, methodology. **Shuvojit Mitra:** investigation, methodology. **Md Bashir Uddin:** validation, visualization, writing – review and editing. **Md. Mukter Hossain:** validation, visualization, formal analysis, supervision, writing – review and editing. **Md. Mahfujur Rahman:** conceptualization, data curation, investigation, formal analysis, methodology, software, project administration, resources, supervision, validation, visualization, writing – original draft, writing – review and editing. All authors have read and agreed to the published version of the manuscript.

## Funding

The authors have nothing to report.

## Ethics Statement

The research work has been approved by the Institutional Ethics Committee at Sylhet Agricultural University, Sylhet‐3100, Bangladesh, with Animal Use Protocol #AUP2023022. All the procedures in this study were handled by well‐trained personnel in accordance with the ethical rules and regulations set by the institution. The welfare of the animals included in the experiment was of prime importance.

## Conflicts of Interest

The authors delcare no conflicts of interest.

## Supporting information


**Figure S1:** Representative agarose gel electrophoresis images showing PCR amplification of species‐specific and virulence genes, including (A) *alr* (369 bp) and (D) *stx1* (302 bp) in *Escherichia coli*, and (B) *gyr*A (441 bp) and (C) *rpo*B (108 bp) in *Klebsiella* spp., with a 100‐bp DNA ladder (M) and negative control (NC).
**Figure S2:** Agarose gel electrophoresis profiles illustrating PCR detection of antimicrobial resistance genes in E. coli and Klebsiella pneumoniae, including A: tetA (502 bp), B: sul1 (433 bp), C: aac(3)‐IV (333 bp), D: str(A) (893 bp), E: MultiCaseDHA (997 bp) and MultiCaseMOX (895 bp), F: blaTEM (800 bp) and blaOXA (564 bp) with a 100‐bp DNA ladder (M) and negative control (NC).
**Table S1:** Reaction mixture and thermal cycling condition for molecular detection of different organisms.

## Data Availability

The data that support the findings of this study are available from the corresponding author upon reasonable request.
