# Supplementary material for: Prevalence, Antimicrobial Resistance, and Resistance Gene Profiles of Extended‐Spectrum β‐Lactamase‐Producing Escherichia coli and Klebsiella pneumoniae Isolated From Quails in Sylhet, Bangladesh
Source: Microbiologyopen. 2026 Jun 16;15(3):e70340. doi: 10.1002/mbo3.70340 (PMC13273018; doi:10.1002/mbo3.70340)
Supplement: Supplementary file 1 — Figure S1: Representative agarose gel electrophoresis images showing PCR amplification of species‐specific and virulence genes, including (A) alr (369 bp) and (D) stx1 (302 bp) in Escherichia coli, and (B) gyrA (441 bp) and (C) rpoB (108 bp) in Klebsiella spp., with a 100‐bp DNA ladder (M) and negative control (NC). Figure S2: Agarose gel electrophoresis profiles illustrating PCR detection of antimicrobial resistance genes in E. coli and Klebsiella pneumoniae, including A: tetA (502 bp), B: sul1 (433 bp), C: aac(3)‐IV (333 bp), D: str(A) (893 bp), E: MultiCaseDHA (997 bp) and MultiCaseMOX (895 bp), F: blaTEM (800 bp) and blaOXA (564 bp) with a 100‐bp DNA ladder (M) and negative control (NC). Table S1: Reaction mixture and thermal cycling condition for molecular detection of different organisms. [file MBO3-15-e70340-s001.docx]

| **Organism** | **Thermal cycle** | **Reaction mixture (25 μl)** |
| --- | --- | --- |
| *E coli* | - **I**nitial denaturation at 94°C for 5 minutes - 35 cycles of denaturation at 95°C for 60 seconds, annealing for 30 seconds at 57°C, and elongation at 72°C for 2 minutes - Final elongation at 72°C for 10 minutes | - 2x master mix (Add Bio Inc, South Korea) at 12.5 μl - 1 μl per primer (forward and reverse) at a concentration of 10 pmol/L - Template DNA 5 μl - Nuclease-free water 5.5 μl |
| Genus: *Klebsiella &*  *Klebsiella pneumoniae* | - Initial denaturation at 95°C for 5 minutes - 35 cycles of denaturation at 95°C for 60 seconds, annealing for 60 seconds at 55°C, and elongation at 72°C for 2 minutes - Final elongation at 72°C for 10 minutes | - 2x master mix (Add Bio Inc, South Korea) at 12.5 μl - 0.5 μl per primer (forward and reverse) at a concentration of 10 pmol/L - Template DNA 5 μl - Nuclease-free water 6.5 μl |
| *Klebsiella oxytoca* | - Initial denaturation for 2 minutes at 95°C - 35 cycles of denaturation for 20 seconds at 94°C, annealing for 20 seconds at 59°C, and elongation at 72°C for 30- seconds - Final elongation at 72°C for 10 minutes | - 2x master mix (Add Bio Inc, South Korea) at 12.5 μl - 0.5 μl per primer (forward and reverse) at a concentration of 10 pmol/L - Template DNA 5 μl - Nuclease-free water 6.5 μl |
| *Stx1* | - Initial denaturation for 2 minutes at 94°C - 35 cycles of denaturation for 1 minute at 94°C, annealing for 1 minute at 55°C, and elongation at 72°C for 1 minute - Final elongation at 72°C for 10 minutes | - 2x master mix (Add Bio Inc, South Korea) at 12.5 μl - 0.5 μl per primer (forward and reverse) at a concentration of 10 pmol/L - Template DNA 5 μl - Nuclease-free water 6.5 μl |
| *blaTEM, blaSHV, blaOXA, blaCTXM1, blaCTXM2, blaCTXM9, MulticaseACC, MulticaseMOX, MulticaseDHA* | - Initial denaturation for 5 minutes at 95°C - 30 cycles of denaturation for 30 seconds at 94°C, annealing for 90 seconds at 62°C, and elongation at 72°C for 1 minute - Final elongation at 72°C for 10 minutes | - 2x master mix (Add Bio Inc, South Korea) at 12.5 μl - 0.5 μl per primer (forward and reverse) at a concentration of 10 pmol/L - Template DNA 5 μl - Nuclease-free water 6.5 μl |
| Gentamicin resistant gene *aac (3)-iv* | - Initial denaturation for 10 minutes at 94°C - 35 cycles of denaturation for 60 seconds at 94°C, annealing for 1 minute at 63°C, extension at 72°C for 1 minute - Final extension at 72°C for 10 minutes | - 2x master mix (Add Bio Inc, South Korea) at 12.5 μl - 1 μl per primer (forward and reverse) at a concentration of 10 pmol/L - Template DNA 5 μl - Nuclease-free water 5.5 μl |
| Sulphonamide resistant gene *Sul1* | - Initial denaturation for 15 minutes at 95°C - 30 cycles of denaturation for 60 seconds at 95°C, annealing for 1 minute at 66°C, extension at 72°C for 1 minute - Final extension at 72°C for 10 minutes | - 2x master mix (Add Bio Inc, South Korea) at 12.5 μl - 1 μl per primer (forward and reverse) at a concentration of 10 pmol/L - Template DNA 5 μl - Nuclease-free water 5.5 μl |
| Tetracycline resistant gene *tetA*, Streptomycin resistant gene *strA* | - Initial denaturation at 94°C for 15 minutes, - 30 cycles of 1 minute at 94°C for denaturation,   1 minute at 63°C for primer annealing, 1minute at 72°C for strand elongation   - Final elongation at 72°C for 10 minutes | - 2x master mix (Add Bio Inc, South Korea) at 12.5 μl - 0.5 μl per primer (forward and reverse) at a concentration of 10 pmol/L - Template DNA 5 μl - Nuclease-free water 5.5 μl |

**Supplementary Table 1:** Reaction mixture and thermal cycling condition for molecular detection of different organisms.


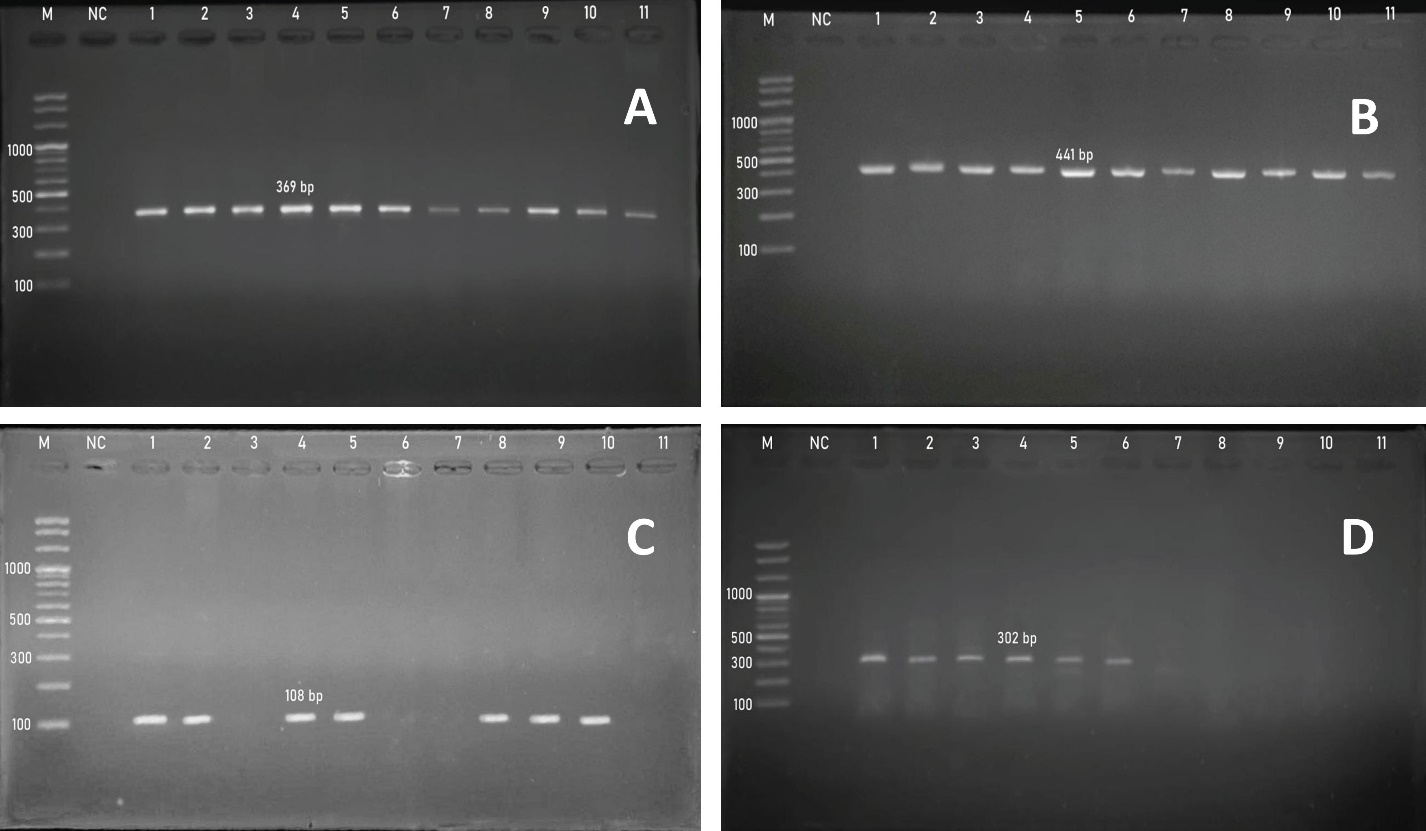


**Supplementary Figure 1:** Representative agarose gel electrophoresis images showing PCR amplification of species-specific and virulence genes, including (A) *alr* (369 bp) and (D) *stx1* (302 bp) in *Escherichia coli*, and (B) *gyrA* (441 bp) and (C) *rpoB* (108 bp) in *Klebsiella* spp., with a 100-bp DNA ladder (M) and negative control (NC).


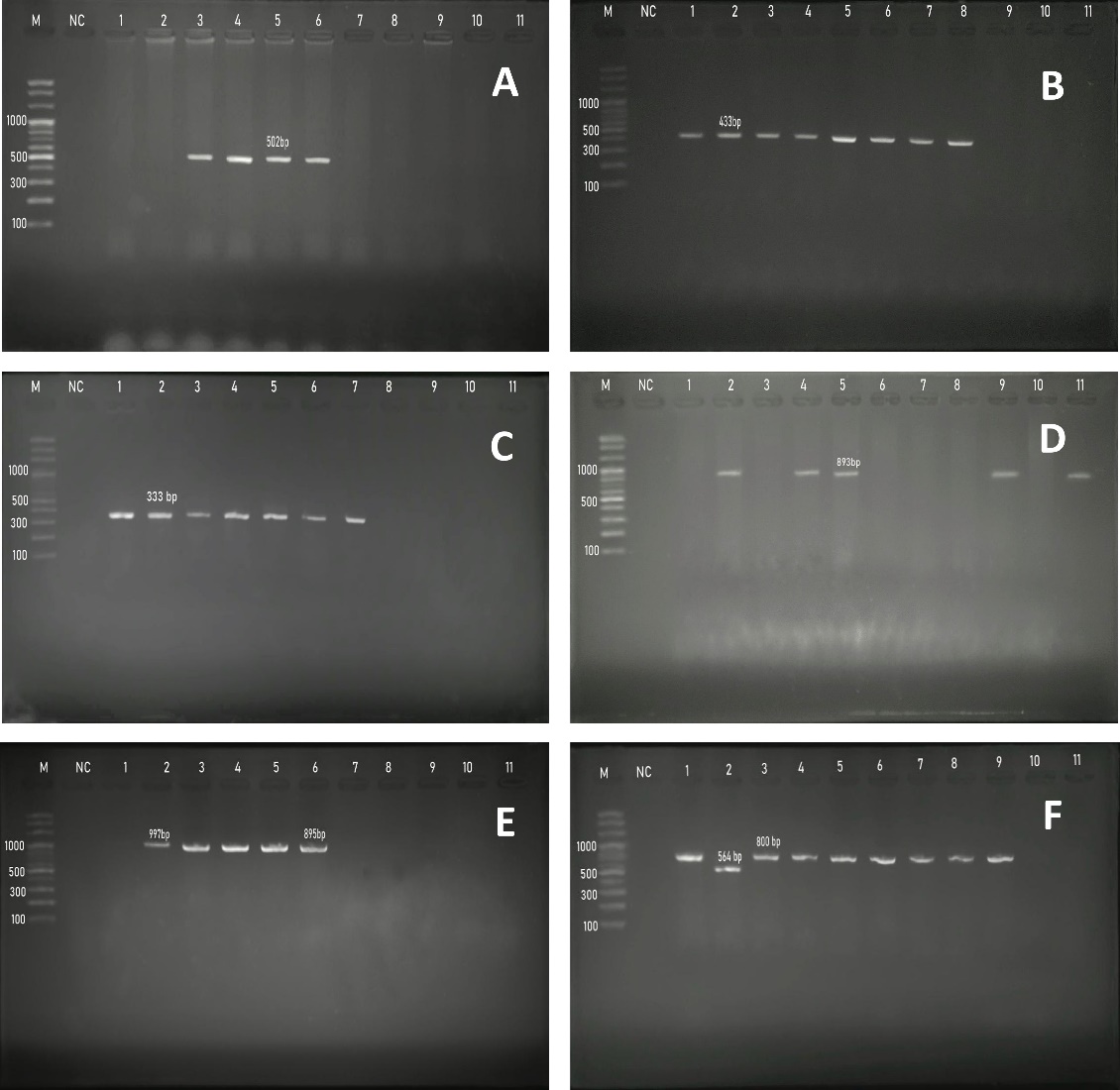


**Supplementary Figure 2:** Agarose gel electrophoresis profiles illustrating PCR detection of antimicrobial resistance genes in *E. coli* and *Klebsiella pneumoniae*, including A: *tetA* (502 bp), B: *sul1* (433 bp), C: *aac(3)-IV* (333 bp), D: *str(A)* (893 bp), E: *MultiCase*_DHA_ (997 bp) and *MultiCase*_MOX_ (895 bp), F: *bla*_TEM_ (800 bp) and *bla*_OXA_ (564 bp) with a 100-bp DNA ladder (M) and negative control (NC).
